# Prosthodontic Rehabilitation as Essential Care Within India’s District Health System: A Narrative Review and Conceptual Framework

**DOI:** 10.1155/ijod/3425087

**Published:** 2026-09-20

**Authors:** Umesh Y. Pai, Aradhana Nagarsekar, Ridhima Gaunkar, Prajakta R. Volvaikar

**Affiliations:** ^1^ Department of Prosthodontics, Manipal College of Dental Sciences Mangalore, Manipal Academy of Higher Education, Manipal, Karnataka, 575001, India, manipal.edu; ^2^ Department of Prosthodontics, Goa Dental College and Hospital, Goa University, Rajiv Gandhi Medical Complex, Bambolim 403202, Goa, India, goa.gov.in; ^3^ Manipal College of Dental Sciences, Mangalore, Manipal Academy of Higher Education (MAHE), Manipal 575001, Karnataka, India, manipal.edu; ^4^ Department of Public Health Dentistry, Goa Dental College and Hospital, Goa University, Rajiv Gandhi Medical Complex, Bambolim 403202, Goa, India, goa.gov.in

**Keywords:** conceptual framework, dentures, district health system, edentulism, essential care, healthy ageing, India, narrative review, NOHP, oral health policy, prosthodontics, universal health coverage

## Abstract

**Background:**

Despite the National Oral Health Programme (NOHP) mandating the provision of removable partial and complete dentures (CDs) at the district level, prosthodontic services in public facilities remain largely inaccessible across most Indian states, even after more than a decade of policy implementation. With India’s elderly population projected to reach 347 million by 2050 and complete edentulism affecting 12%–14% of older adults, the unmet need for prosthodontic care is substantial and continues to grow.

**Aim:**

This narrative review synthesises available evidence on the burden and impact of edentulism, along with policy responses in India and comparable settings. It further proposes a conceptual framework for essential prosthodontic care within India’s district health system (DHS), positioning prosthodontic rehabilitation as a core health service, comparable to cataract surgery and hearing rehabilitation.

**Methods:**

A narrative review was conducted following scale for the assessment of narrative review articles (SANRA) guidelines, drawing on epidemiological data, policy documents, low‐ and middle‐income country (LMIC) programme evaluations and health economics evidence.

**Synthesis and Proposed Framework:**

Nine critical implementation gaps were identified from the literature. In response, the DHS Essential Prosthodontic Services Framework is proposed, comprising a five‐tier service package with the district hospitals as nodal fabrication units; a hub‐and‐spoke laboratory model; eligibility and benefit structure aligned with PM‐JAY; integration with National Programme for Health Care of the Elderly (NPHCE) and Ayushman Bharat; eight key performance indicators and a phased 10‐year implementation roadmap.

**Conclusion:**

Framing prosthodontic rehabilitation as essential care comparable to cataract surgery provides the most practical and sustainable basis for the administrative reforms required to translate existing policy intent into functional district‐level service delivery.

## 1. Introduction

Tooth loss, the cumulative and irreversible outcome of dental caries, periodontitis and trauma represents a significant yet under‐recognised public health challenge in India. It disproportionately affects older adults, rural populations and socioeconomically disadvantaged groups who have limited access to preventive and restorative dental care and for whom private prosthodontic services remain largely unaffordable. Tooth loss is well established to result in functional impairment, compromised nutrition and a diminished quality of life. Despite this, the overall burden of edentulism has remained relatively unchanged over time, reflecting persistent gaps in prevention, early intervention and equitable service delivery [1, 2]. In the context of a rapidly ageing population, this underscores the need for more robust and systemic health system responses [3].

Untreated edentulism has been associated with broader systemic consequences, including impaired mastication, increased risk of sarcopenia and frailty, as well as cognitive decline and social isolation [4, 5]. Importantly, the long‐term economic burden arising from these sequelae may exceed the costs associated with timely prosthodontic rehabilitation. Evidence from international public health data suggests that the per‐case cost of complete denture (CD) fabrication in public sector settings falls within widely accepted cost‐effectiveness thresholds for public financing [6].

In India, the National Oral Health Programme (NOHP), updated in 2024 under the National Health Mission, mandates the provision of basic prosthetic rehabilitation such as removable partial dentures (RPDs) and CDs at district and sub‐district dental facilities. However, implementation remains inconsistent across states. Key barriers include inadequate dental laboratory infrastructure, shortages of trained dental technicians, the absence of dedicated budget allocations for dental materials and weak referral mechanisms [7].

In this context, the present narrative review provides evidence on the impact of edentulism and evaluates current policy responses in India and comparable health systems. Furthermore, it proposes a conceptual framework to integrate prosthodontic care within India’s district health system (DHS), positioning prosthodontic rehabilitation as an essential health service comparable in priority to cataract surgery and hearing rehabilitation.

## 2. Methods

A narrative review was undertaken in accordance with established methodological guidance, including the scale for the assessment of narrative review articles (SANRA) [8]. A comprehensive literature search was conducted independently by two authors between January 2026 and February 2026. Literature published between 2000 and 2026 was included. A literature search was conducted across multiple electronic databases, including PubMed, MEDLINE, Web of Science, Scopus, ScienceDirect and Google Scholar. This was supplemented by manual searches of relevant peer‐reviewed journals, as well as grey literature sources such as publications from the World Health Organization (WHO) and official policy documents from the Ministry of Health and Family Welfare and the National Health Mission, Government of India.

The literature search was conducted using a combination of key words such as oral health policy in India, prosthodontic care, denture services, edentulism, geriatric oral health and access to dental care. A focused search process was subsequently applied to explore key domains: population ageing, burden of edentulism, systemic and quality of life impacts of tooth loss, gaps in public prosthodontic service delivery, workforce and infrastructure limitations, financial protection mechanisms and integration of oral health within national health programmes such as Ayushman Bharat and the National Programme for Health Care of the Elderly (NPHCE).

Relevant evidence was synthesised from national and international sources, including NOHP policy documents, National Family Health Survey (NFHS‐5), Global Burden of Disease (GBD) 2017 data and the Disease Control Priorities project. Global policy frameworks from the FDI World Dental Federation and evaluations of public prosthodontic programmes in countries such as Brazil, Sri Lanka, Thailand and Chile were also reviewed to provide comparative and contextual insights.

The review followed predefined inclusion and exclusion criteria as follows:

Inclusion:

• Manuscripts published in peer‐reviewed journals.
• Policy documents, government reports and programme guidelines related to oral health and prosthodontic care in India.
• Studies addressing edentulism, prosthodontic rehabilitation and health system responses in India and comparable settings.

Exclusion:

• Studies focusing primarily on clinical dental procedures without policy or public health relevance.
• Studies addressing non‐geriatric priority populations without relevance to edentulism or prosthodontic care.

This study is a narrative review based exclusively on the published literature, policy documents and publicly available sources. No human participants, patient data, identifiable personal information or intervention were involved. Therefore, ethical approval and informed consent were not required.

## 3. Discussion

### 3.1. Epidemiological Burden of Edentulism in India

Data from the National Oral Health Survey indicate that edentulism affects 11.5% of the urban elderly population and 13.0% of their rural counterparts, with severe partial edentulism demonstrating an even higher prevalence [2, 9, 10]. Findings from the NFHS‐5 (2019–2021) suggest minimal improvement in these indicators despite the policy directives outlined under the NOHP. Furthermore, the GBD 2017 study identifies severe tooth loss as a leading contributor to years lived with disability among adults aged over 50 years in India [1].

These epidemiological trends must be interpreted in the context of rapid population ageing. In 2022, India had an estimated 149 million individuals aged 60 years and above, a figure projected to increase to 347 million by 2050, surpassing the adolescent population [3]. This demographic transition underscores the urgency for targeted health system responses. Even under conservative prevalence estimates, these projections imply that tens of millions of older adults will require prosthodontic rehabilitation in the coming decades. The existing public health infrastructure; however, remains inadequately equipped to address this growing demand, highlighting a substantial service delivery gap.

### 3.2. Functional, Nutritional and Psychosocial Consequences of Edentulism

Research by Chan et al. [5] found that adults with fewer functional teeth ate less varied and lower‐quality diets, with reduced protein and fibre intake. Prosthodontic rehabilitation in older adults enhances masticatory function and quality of life, with modest yet consistent improvements in nutritional outcomes [11].

According to the WHO Global Oral Health Status Report 2022, oral diseases affect 3.5 billion people globally and severe tooth loss is a leading contributor to disability‐adjusted life years in low‐ and middle‐income countries (LMICs) [12]. Beyond nutrition, tooth loss is independently associated with cognitive decline, social isolation, depression and reduced quality of life in older adults [4]. The long‐term costs resulting from these outcomes: hospitalisation, malnutrition treatment and frailty‐related disability care, significantly exceed the expenses associated with prosthodontic rehabilitation [6].

### 3.3. Prosthodontic Rehabilitation as Essential Care: the Policy Argument

Providing basic removable and fixed prostheses to restore oral function after tooth loss is recognised as essential care by the WHO Universal Health Coverage (UHC) Framework and the Disease Control Priorities project [13, 14]. A service qualifies as essential if it: (i) tackles a high‐burden, equity‐relevant health need; (ii) is cost‐effective by established thresholds; (iii) has a generalisable evidence base; (iv) is committed to in national or international health policy and (v) requires systematic, financed and monitored delivery within the public health sector to guarantee fair access [13]. Basic prosthodontic rehabilitation satisfies all five criteria without ambiguity.

Prosthodontic rehabilitation is increasingly recognised as a cost‐effective intervention within oral health strategies for LMICs [6]. Global policy frameworks have reinforced this position. The WHO Global Oral Health Action Plan 2023–2030 [15] and the World Health Assembly resolution WHA74.5 (2021) emphasise the inclusion of essential oral health services, including prosthodontic rehabilitation for edentulous populations, within UHC frameworks [16, 17]. Similarly, the FDI World Dental Federation’s Vision 2030 underscores tooth replacement as an integral component of comprehensive oral healthcare rather than an elective service, highlighting that failure to provide such care to vulnerable populations constitutes inequitable access based on socioeconomic status [18].

Further supporting this perspective, the third edition of the disease control priorities identifies basic prosthodontic rehabilitation as a priority intervention for LMIC health systems, citing its favourable cost‐effectiveness profile and potential to improve equity in health outcomes [14].

At the national level, India’s public health policies, including the NOHP and Ayushman Bharat, formally acknowledge the need to provide prosthodontic rehabilitation for populations unable to access private care [15]. However, translating these policy commitments into effective service delivery remains a critical challenge. An essential care framework is therefore required to operationalise these commitments through strengthened infrastructure, an adequately trained workforce, sustainable financing mechanisms and robust monitoring systems to ensure efficient delivery of prosthodontic services.

### 3.4. Policy Gaps and the Implementation Deficit

The operational guidelines of the NOHP, updated in 2024 under the National Health Mission, explicitly mandate the provision of RPDs and CDs at district‐level dental facilities [15]. Despite this clear policy directive, implementation across most Indian states remains limited, reflecting a substantial disconnect between policy intent and service delivery. Synthesis of the available literature highlights several critical gaps such as:

1. Rapid expansion of the ageing population, coupled with a persistent burden of edentulism, presents a demand that the current health system is inadequately prepared to address [1, 3].
2. Functional, nutritional and psychosocial consequences of untreated tooth loss contribute significant downstream health and economic burdens, further amplifying the need for timely prosthodontic intervention [4–6].
3. Limited prosthodontic services due to persistent gaps in NOHP implementation across most Indian states [7, 15].
4. The lack of dedicated dental laboratory infrastructure and laboratory support at the district level [7].
5. Shortage of trained dental technicians and absence of sanctioned workforce positions within the National Health Mission [7].
6. Financial barriers and limited coverage for denture services under existing health financing schemes [6, 19].
7. The lack of integration of oral health within broader geriatric health programmes including NPHCE [20].
8. Inequitable access to prosthodontic care across regions, with rural and tribal populations disproportionately affected [9, 10].
9. Absence of a structured, monitored service delivery framework for prosthodontic care at the district level [7, 15].

The barrier to implementation is largely administrative rather than structural. India already produces trained dental technicians through DCI‐accredited programmes, including the 3‐year Bachelor of Science in Dental Technology (B.Sc Dental Technology) and the 2‐year Diploma in Dental Mechanics, offered through various government and private institutions. However, the lack of designated positions within the National Health Mission prevents their integration into public services [7]. Addressing this requires only an NHM notification and not new legislation. Similarly, including RPDs and CDs in the PM‐JAY benefit package at public sector rates of approximately ₹2500–5000 per case, which is significantly below the private sector cost of approximately ₹8000–25,000, requires only an administrative notification from the National Health Authority.

The National Programme for Control of Blindness provides a relevant precedent [21]. Cataract surgery became an integrated, essential part of the health system with posts created, resources allocated, training mandated and outcomes tracked. Denture provision requires an identical transition from a mandated but undelivered service to one that is properly resourced, staffed, monitored and provided at scale.

### 3.5. International Evidence: What LMIC Settings Have Demonstrated

Examples from comparable settings demonstrate that large‐scale public provision of dentures is feasible in LMICs. Brazil’s National Oral Health Policy (Brasil Sorridente) expanded access to prosthodontic rehabilitation through a network of Family Health Care Strategy of the Dentistry Specialisation Centres (CEO) and Regional Dental Prosthesis Laboratories (LRPD), demonstrating how public health systems can integrate denture services into routine oral healthcare delivery [22]. Sri Lanka has strengthened access to prosthodontic care through the expansion of government dental services, including the establishment of dental laboratories in district and provincial hospitals. This approach has improved the availability of prosthodontic rehabilitation services, particularly for rural and underserved populations, demonstrating the value of integrating dental laboratory infrastructure within the public health system [23].

In Thailand, the benefits of dental prosthetics are significantly supported through the Royal Thai Denture Project and the National Health Security Office (NHSO), which provide free or subsidised dental prosthetics to low‐income population [24].

The WHO Global Oral Health Action Plan 2023–2030 identifies Strategic Objective 4 as the integration of essential oral health services, including the management of tooth loss into primary healthcare systems and UHC benefit packages [16]. This strategic direction provides a coherent global policy framework within which India’s proposed district‐level prosthodontic care model can be situated, reinforcing the need to imbibe prosthodontic rehabilitation within accessible public health services.

### 3.6. A Proposed Conceptual Framework Principles for Prosthodontic Care Integration

First, the essential care principle: recognises prosthodontic rehabilitation as a fundamental health need rather than a purely cosmetic or specialist intervention, consistent with national policy under the NOHP [15] as well as global directives from the WHO [16].

Second, the DHS integration principle: prosthodontic services should be incorporated into existing health system infrastructure: referral pathways, workforce structures, supply chains and financing, rather than creating parallel systems.

Third, the district hospital nodal principle: district hospitals are the most appropriate and feasible level for denture fabrication as they can sustain laboratory infrastructure, maintain dental technician positions with adequate workloads and provide specialist supervision. Lower‐level facilities focus on patient screening, referral and follow‐up.

### 3.7. Tiered Service Package

The framework defines a five‐tier service package with distinct responsibilities at each level. Peripheral levels (Tiers 1–3) handle identification, referral and follow‐up. District hospitals (Tier 4) are the designated nodal fabrication units responsible for comprehensive assessment, prosthesis fabrication and delivery. Tertiary centres (Tier 5) manage complex rehabilitation, advanced digital fabrication and specialist training. This division between clinical and technical roles reflects the core design feature of effective public prosthodontic programmes in Brazil, Sri Lanka and Thailand [22–24]. Table 1 presents the complete tiered delivery framework.

**Table 1:** DHS essential prosthodontic services package—tiered delivery framework.

Level	Facility	Essential prosthodontic services	Workforce	Infrastructure
1	Ayushman Arogya Mandir/sub‐centre	Edentulism screening; referral card; denture hygiene counselling; follow‐up support	CHO; ASHA (oral health module trained)	IEC materials; referral register; denture hygiene supply kit
2	Primary health centre	Dental examination; edentulism registration; pre‐prosthetic oral care; simple extractions; referral to district hospital; post‐delivery review	NOHP‐posted dental officer	Basic dental chair; extraction instruments; impression trays; referral tracking system
3	Community health centre	Comprehensive oral examination; prosthodontic treatment planning; preliminary impressions; jaw relation; denture delivery and review	Dental officer with prosthodontic training; visiting dental technician	Dental unit; with prosthetic inventory and laboratory equipments
4 (NODAL)	District hospital	Full essential package: complete dentures; RPDs; simple FPDs; overdentures; immediate dentures; relines and repairs; pre‐prosthetic minor surgery	Dental officer; visiting prosthodontist; dental technician (sanctioned NHM post); dental nurse	Dental laboratory (on‐site OR hub‐linked); CAD/CAM and intraoral scanner
5	Dental college/tertiary centre	Complex rehabilitation; implant prosthetics; maxillofacial prosthetics; digital fabrication hub; training; quality assurance; research	Prosthodontist faculty; dental technologist; research staff	Advanced digital lab; CAD/CAM hub; teleconsultation platform

*Note:* The district hospital (Level 4) is the designated nodal fabrication unit. Levels 1–3 perform patient‐facing functions only.

Abbreviations: ASHA, accredited social health activist; CAD/CAM, computer‐aided design/manufacturing; CHO, community health officer; FPD, fixed partial denture; NOHP, National Oral Health Programme; RPD, removable partial denture.

### 3.8. Hub‐and‐Spoke Laboratory Model

The largest operational challenge to providing prosthodontic services at the district level is the absence of functional dental laboratories in most district hospitals [7]. The hub‐and‐spoke model addresses this by centralising fabrication infrastructure at hub sites, dental colleges or regional dental centres, while allowing all patient‐facing clinical steps at spoke sites: district hospitals and CHCs. In Phase 1, conventional impression materials and physical transport are used with a target turnaround of five working days and a 21‐day clinical delivery goal. In Phase 2, digital intraoral scanners at spoke sites enable electronic file transfer to hubs, reducing the turnaround to 14 days or less [25]. Phase 3 introduces 3D printing at hub sites, lowering per‐unit material costs and creating economies of scale. Digital workflow should be conceptualised as a phased enhancement rather than a prerequisite for prosthodontic service initiation without a delay due to technological dependencies.

The successful implementation of the proposed hub‐and‐spoke model will depend on adequate training and capacity building among dental professionals and laboratory technicians. In the initial phases, tertiary hospitals and dental colleges will function as training and resource centres, providing hands‐on training, technical support and mentorship to dental officers and dental technicians in digital dentistry workflows, thereby facilitating smooth adoption of new technologies within DHSs. Emphasis will be placed on structured training programmes and continuing professional development activities focused on intraoral scanning, computer‐aided design and laboratory communication. A gradual transition may facilitate technology adoption and minimise resistance to change.

### 3.9. Eligibility and Benefit Package

The proposed framework provides an outline for a structured eligibility and benefit package aligned with India’s existing social protection architecture [19].

At its core, Priority Group 1 includes below‐poverty‐line edentulous adults aged 60 years and above, who are eligible for fully subsidised denture services. This group reflects both the highest level of clinical need andan equity‐based justification for public financing.

Subsequent tiers (priority Groups 2 and 3) enable a phased expansion of coverage, contingent upon the availability of financial resources and strengthening of service delivery infrastructure. Table 2 presents the complete eligibility, entitlement and financing structure.

**Table 2:** Essential prosthodontic benefit package—eligibility, entitlement and financing.

Priority group	Clinical eligibility	Entitlement	Financing mechanism
Group 1—fully subsidised	Complete edentulism (≥1 arch); age ≥60 years; BPL/PM‐JAY beneficiary	Complete dentures (both arches); one reline per 5 years; repairs covered; replacement at 8 years	PM‐JAY benefit package; NOHP NHM state allocation; social welfare convergence
Group 2—subsidised	Severe partial edentulism (≥6 missing posterior teeth per arch); age ≥45 years; income ≤₹2 lakh/year	Removable partial dentures (acrylic or metal framework); one reline/repair per 5 years; co‐payment ≤10%	PM‐JAY benefit package expansion; state health insurance schemes; nominal regulated co‐payment
Group 3—cost recovery	Single or multiple missing teeth with documented functional impairment; any age; APL population	RPD or simple FPD; professional services subsidised; materials at cost; user fee ≤₹500; waived for SC/ST and persons with disability	NOHP facility revenue; nominal user fee
Special provisions	Post‐oncologic oral defect; cleft palate; maxillofacial trauma; congenital anomaly	Obturators; maxillofacial prostheses; implant‐retained prostheses where indicated	Rashtriya Arogya Nidhi; state discretionary health fund; tertiary referral

Abbreviations: APL, Above poverty line; BPL, below poverty line; FPD, fixed partial denture; NHM, National Health Mission; PM‐JAY, Pradhan Mantri Jan Arogya Yojana; RPD, removable partial denture; SC/ST, scheduled caste/scheduled tribe.

### 3.10. Integration With Existing National Programmes

The framework aligns with three existing national programmes at the district level. Under NOHP and NHM [15], it calls for designation of dental technician posts, inclusion of prosthodontic materials in central NHM procurement lists, updated facility inspection criteria and integration of prosthodontic indicators into HMIS. Under NPHCE [20], edentulism screening and functional oral assessments are incorporated into the comprehensive geriatric assessment (CGA) at district hospital geriatric OPDs, immediately actionable as both services are co‐located and serve the highest‐need population. Under Ayushman Bharat Health and Wellness Centres [19], Community Health Officers and ASHAs can screen for edentulism during household visits and NCD camps, provide referral cards and support follow‐up and promote awareness regarding the availability and benefits of prosthodontic rehabilitation services. Individuals identified with unmet prosthodontic needs may then be referred through established healthcare pathways for further assessment and treatment. Such integration would enable existing community‐based health networks under Ayushman Bharat and NPHCE to serve as an initial point of contact, thereby improving service awareness, early identification of need and utilisation of prosthodontic care among underserved populations.

### 3.11. Monitoring and Evaluation Framework

Eight key performance indicators are proposed, covering access, equity, quality, timeliness, financial protection, workforce, clinical outcomes and nutritional impact [26]. Each is linked to a specific data source and a 5‐year target. A prosthodontic service register at each district hospital dental unit, integrated into the existing NOHP‐HMIS reporting system, provides the monitoring infrastructure without requiring a separate database. Table 3 presents the complete monitoring and evaluation framework.

**Table 3:** Monitoring and evaluation framework—key indicators, data sources and targets.

Domain	Indicator	Data source	5‐year target
Access	Proportion of eligible edentulous adults (age ≥60, BPL) receiving dentures through DHS in programme districts	Prosthodontic service register at DH	≥30% of estimated need met
Equity	Rural‐urban gap in denture coverage rate within programme states	Prosthodontic service register at DH	Gap reduced 50% from baseline
Quality	Mean GOHAI score change from pre‐treatment to 3‐month post‐delivery	Pre Treatment survey and 3‐month follow‐up	Mean GOHAI improvement ≥8 points
Timeliness	Median days from identification to prosthesis delivery	Prosthodontic service register at DH	≤21 days conventional; ≤14 days digital hub model
Financial protection	Out‐of‐pocket expenditure per group 1 eligible patient	Household survey	<₹200 per patient (group 1); <₹500 (group 2)
Workforce	District hospitals with functioning prosthodontic service (dental officer + dental technician + laboratory)	Directorate of health services record	100% of district hospitals in programme states by year 5
Clinical outcome	Prostheses with satisfactory fit at 3‐month review (no reline required within 3 months)	Clinical review register	≥85% satisfactory fit
Nutritional impact	Increase in MNA‐SF score from pre‐treatment to 6 months post‐delivery in elderly recipients	Prospective cohort sub‐study in 2 sentinel districts	Significant improvement

Abbreviations: BPL, below poverty line; DH, district hospital; GOHAI, geriatric oral health assessment index; MNA‐SF, mini nutritional assessment short form.

### 3.12. Phased Implementation Roadmap

Phase 1—Foundation (Years 1–2): Conducted a national situational analysis in five pilot states using an adapted WHO SARA tool to create a district prosthodontic readiness index (DPRI) for prioritisation. The essential service package was validated through Delphi, a unit cost study was carried out, national prosthodontic service standards were draughted for the NOHP and 10 district hospitals were designated as Phase 1 nodal centres. These hospitals will establish dental technician posts, procure laboratory equipment and deliver a 4‐week prosthodontics training module for dental officers.

Phase 2—Pilot and Learn (Years 2–4): Rolled out services at the 10 nodal district hospital centres and their linked CHCs. Recruited a pilot cohort via NPHCE geriatric OPD referrals and AAM screening and collected GOHAI, MNA‐SF, process time and cost and compliance data with quarterly reviews [26]. A digital hub‐and‐spoke pilot was initiated at two dental colleges and district hospitals and prosthodontic indicators were integrated into HMIS in all pilot districts.

Phase 3—Scale‐Up (Years 4–7): Expanded to all district hospitals in pilot states, guided by Phase 2 evidence. Notified the PM‐JAY benefit package for RPD and CD with rates set from Phase 2 cost data [19], included budget lines in state PIPs, extended task‐sharing to CHOs and ANMs for denture maintenance and rolled out the digital hub‐and‐spoke model across all dental college and district hospital pairs [25].

Phase 4—Nationalisation (Years 7–10): Issued a policy notification under NOHP making prosthodontic services a mandated essential district‐level service [15]. Set national accreditation standards for DHS dental laboratories, conducted a national survey of edentulism and prosthodontic coverage and formally integrated edentulism screening into the NPHCE CGA at every district hospital [20].

### 3.13. Financial Requirements for Implementation

The financial requirements for integrating removable prosthodontic rehabilitation into district health services are expected to be modest relative to other specialised healthcare programmes, as much of the required clinical infrastructure already exists within district hospitals. Initial investments would primarily involve the procurement of prosthodontic instruments, impression materials, basic laboratory equipment and workforce training. Recurring costs would largely comprise laboratory materials, maintenance and personnel support. However, the actual unit cost of service delivery may vary across regions, depending on the workforce availability, laboratory support, patient volume and material requirements. Therefore, pilot studies conducted during the phased implementation of the programme should include a detailed cost analysis to determine the unit cost of prosthodontic rehabilitation. These findings would provide important evidence for future budget allocation, reimbursement planning under PM‐JAY and large‐scale implementation within the DHS.

## 4. Limitations

The methodology used, synthesising policy documents, epidemiological data, LMIC programme comparators and health economic evidence, is appropriate for a conceptual framework but has inherent limitations. This review synthesised on published literature and policy documents and does not incorporate primary data on implementation feasibility across diverse state contexts in India. Consequently, essential operational considerations such as the acceptability of the proposed framework among district health administrators, supply chain constraints and context‐specific accessible barriers, particularly among the elderly rural population, remain to be empirically evaluated. These aspects warrant focused primary research prior to subsequent phases of programme rollout.

A further limitation is that the proposed framework partially draws on the experiences from other LMICs. Given the differences in health system structure, economic resources, population demographics and cultural factors, these models may not be directly transferable to the Indian context and will require adaptation and validation through local situational analyses, pilot implementation and feasibility assessments prior to wider scale‐up. As a narrative review, the possibility of selection bias in the identification and interpretation of evidence cannot be entirely excluded. Adherence to the SANRA guidelines enhanced the methodological rigour of the review and reduced the risk of selection bias [8].

## 5. Conclusion

Edentulism in India remains a significant public health issue with extensive functional, nutritional and psychosocial effects that are not sufficiently addressed. Although policies and global standards exist, the absence of a practical framework has hindered proper service delivery. Viewing prosthodontic rehabilitation as a vital part of UHC offers a realistic solution to this challenge. The suggested DHS approach presents a practical, equity‐focused model to incorporate prosthodontic services into the current health infrastructure. Through administrative focus and strategic action, India can move from policy acknowledgement to tangible results, making restoration of oral function a standard, accessible care instead of an unmet need.

## Author Contributions

**Umesh Y. Pai:** conceptualisation, methodology, writing – original draft, investigation. **Aradhana Nagarsekar:** conceptualisation, investigation, data curation, writing – original draft, validation, supervision. **Ridhima Gaunkar:** data curation, supervision, validation, writing – review and editing. **Prajakta R. Volvaikar:** project administration, supervision, validation, writing – review and editing.

## Funding

This study did not receive any specific funding.

## Disclosure

All authors have read and approved the final version of the manuscript. Aradhana Nagarsekar had full access to all of the data in this study and takes complete responsibility for the integrity of the data and the accuracy of the data analysis.

## Consent

The authors have nothing to report.

## Conflicts of Interest

The authors declare no conflicts of interest.

## Data Availability

Data sharing is not applicable as no new data were generated, the article describes entirely theoretical research.
